# Deep sequencing analyses expands the *Pseudomonas aeruginosa* AmpR regulon to include small RNA-mediated regulation of iron acquisition, heat shock and oxidative stress response

**DOI:** 10.1093/nar/gkt942

**Published:** 2013-10-23

**Authors:** Deepak Balasubramanian, Hansi Kumari, Melita Jaric, Mitch Fernandez, Keith H. Turner, Simon L. Dove, Giri Narasimhan, Stephen Lory, Kalai Mathee

**Affiliations:** ^1^Department of Biological Sciences, College of Arts and Science, Florida International University, Miami, FL 33199, USA, ^2^Department of Molecular Microbiology and Infectious Diseases, Herbert Wertheim College of Medicine, Florida International University, Miami, FL 33199, USA, ^3^BioRG, School of Computing and Information Science, College of Engineering and Computing, Florida International University, Miami, FL 33199, USA, ^4^Division of Infectious Diseases, Boston Children’s Hospital, Harvard Medical School, Boston, MA 02115, USA and ^5^Department of Microbiology and Immunobiology, Harvard Medical School, Boston, MA 02115, USA

## Abstract

Pathogenicity of *Pseudomonas aeruginosa*, a major cause of many acute and chronic human infections, is determined by tightly regulated expression of multiple virulence factors. Quorum sensing (QS) controls expression of many of these pathogenic determinants. Previous microarray studies have shown that the AmpC β-lactamase regulator AmpR, a member of the LysR family of transcription factors, also controls non-β-lactam resistance and multiple virulence mechanisms. Using RNA-Seq and complementary assays, this study further expands the AmpR regulon to include diverse processes such as oxidative stress, heat shock and iron uptake. Importantly, AmpR affects many of these phenotypes, in part, by regulating expression of non-coding RNAs such as rgP32, asRgsA, asPrrF1 and rgRsmZ. AmpR positively regulates expression of the major QS regulators LasR, RhlR and MvfR, and genes of the *Pseudomonas* quinolone system. Chromatin immunoprecipitation (ChIP)-Seq and ChIP–quantitative real-time polymerase chain reaction studies show that AmpR binds to the *ampC* promoter both in the absence and presence of β-lactams. In addition, AmpR directly binds the *lasR* promoter, encoding the QS master regulator. Comparison of the AmpR-binding sequences from the transcriptome and ChIP-Seq analyses identified an AT-rich consensus-binding motif. This study further attests to the role of AmpR in regulating virulence and physiological processes in *P. aeruginosa*.

## INTRODUCTION

*Pseudomonas aeruginosa* is ubiquitous, and can be isolated from diverse sources including plants, animals and humans. A high degree of nutritional versatility and adaptability ensure that *P. aeruginosa* is able to colonize a wide range of natural and man-made habitats. In humans, *P. aeruginosa* is seldom part of the normal microbial flora and is found in <2–6% of individuals (1). An opportunistic, nosocomial pathogen, *P. aeruginosa* colonization rates in hospitalized patients, however, can be >50% and especially so in cases of mucosal or cutaneous breach, or in immunocompromised individuals (2). *Pseudomonas aeruginosa* is also the leading cause of morbidity and mortality in cystic fibrosis (CF) patients (3).

The pathogenic potential of *P. aeruginosa* is multifactorial and can be broadly classified into cell-associated and secreted virulence factors. The cell-associated virulence factors are typically structural components of the cell, such as the lipopolysaccharide, pili and flagella (4–6). The process of quorum sensing (QS) regulates expression of many of the major secreted virulence factors. QS is a mechanism of coordinating gene expression based on the population density, employed by both non-pathogenic and pathogenic bacteria (7). Quoromones (acyl-homoserine lactones) are small diffusible molecules that mediate QS communication between cells to synchronize expression of virulence genes (8). Precise signaling is ensured by the species-specific nature of quoromones, although crosstalk between related bacteria is known to occur (9,10). *Pseudomonas aeruginosa* employs three interdependent mechanisms of QS, namely, the Las, Rhl and *Pseudomonas* quinolone system (PQS). The Las system is at the top of the regulatory hierarchy, above the Rhl system, while PQS interacts with both Las and Rhl [reviewed in (11,12)]. In *P. aeruginosa*, the QS process controls production of secreted enzymes and toxins such as LasA, LasB and ToxA; redox-active compounds such as phenazines (12) and in the case of chronic infections, the formation of bacterial communities called biofilms (13). In addition, some efflux pumps, such as MexGHI-OpmD, which play a role in pumping out quoromones from the cytoplasm to the cell exterior, are also QS-regulated (14,15). However, a majority of the 12 putative and established RND efflux pumps in *P. aeruginosa* are involved in antibiotic resistance (16,17).

Antibiotic resistance is a major problem in dealing with *P. aeruginosa* infections. The current treatment regimen for *P. aeruginosa* is typically a combination therapy of β-lactams, aminoglycosides and quinolones (3,18). However, a 6-year survey by the National Nosocomial Infections Surveillance System of the Centers for Disease Control and Prevention revealed that *P. aeruginosa* isolates were resistant to many commonly used antibiotics in both intensive-care unit and non-intensive-care unit patients (19). The infection rates with antibiotic-resistant *P. aeruginosa* were as high as 36% (19).

*Pseudomonas aeruginosa* has multiple mechanisms of antibiotic resistance (16). Resistance to the β-lactam class of antibiotics is primarily conferred by the chromosomally encoded β-lactamase AmpC (16). The MexEF-OprN efflux pump mediates quinolone resistance (20). Our recent study demonstrated that the LysR-type transcriptional regulator (LTTR) AmpR modulates expression of both *ampC* and *mexEF-oprN* (21). In addition, *P. aeruginosa* AmpR is a global regulator of many virulence determinants and transcriptional factors (21,22). Using DNA microarrays and complementary assays, we have demonstrated that the AmpR regulon consists of >500 genes that are involved in virulence and metabolism (21). Importantly, the analyses reveal that AmpR positively regulates many acute infection phenotypes while repressing chronic ones (21). Interestingly, the AmpR regulon included the small regulatory RNA rgRsmZ (21). Given the extensive nature of the AmpR regulon, we hypothesized that other small regulatory RNAs could have been missed, as the microarray platform is not designed to detect them. In addition, given the limited sensitivity of microarrays, other potentially AmpR-regulated genes may have escaped detection. This study uses RNA-Seq to identify other non-coding RNAs (ncRNAs) and chromatin immunoprecipitation (ChIP)-Seq to determine direct targets of AmpR. Furthermore, we assign a role for AmpR in previously unidentified critical cellular processes such as iron uptake, oxidative stress and heat shock. This study reaffirms AmpR as a critical regulator of *P. aeruginosa* virulence and physiological processes.

## MATERIALS AND METHODS

### Strains, plasmids, primers and culture conditions

The strains and plasmids used in this study are listed in Table 1. The primers used are listed in Supplementary Table S1. The wild-type *P. aeruginosa* PAO1 and its isogenic in-frame *ampR* deletion strain, PAOΔ*ampR*, are described earlier (21,23).

**Table 1.** Strains and plasmids used in this study

Strain/plasmid	Relevant characteristics	Source
Strains		
*Escherichia coli*		
DH5α	General purpose cloning strain; Δ*(lacZ)M15*	New England Biolabs
DBS206	*E. coli* DH5α harboring 3x-V5 tag on pCR2.1 TOPO	This study
DBS215	DH5α with *ampR* ORF and the *ampR-ampC* intergenic region PCR cloned into pCR2.1 TOPO	This study
DBS222	DH5α harboring *ampR* ORF tagged with 3x V5-tag on pCR2.1 TOPO	This study
DBS234	DH5α with mini CTX2, containing 3x V5-tagged *ampR*	This study
*Pseudomonas aeruginosa*		
PAO1	Wild-type	(23)
PKM315	PAOΔ*ampR*; in-frame deletion of *ampR* (*PA4109*)	(21)
DBS248	PAOΔ*ampR*::*ampR*-V5; 3x-V5-tagged *ampR* cloned onto mini-CTX2 and moved into PKM315	This study
Plasmids		
pCR2.1 TOPO	TA cloning vector for PCR products; Ap^R^, Km^R^; ColE1 f1 *ori lacZα*	Invitrogen
ZM747-V5	3x V5 tag C-term and N-term of yeast URA-3 in pBlueScript; pDBS193	(24)
Mini CTX2	Plasmid for single-copy gene integration in *P. aeruginosa*; Tc^R^	(25)
pDBS206	3x V5 tag PCR-amplified from ZM747-V5 and cloned into pCR2.1 TOPO	This study
pDBS215	1112-bp *ampR* ORF along with the *ampR-ampC* intergenic region, PCR-amplified with primers DBS_*ampR*F1 and DBS*ampR*; cloned into pCR2.1 TOPO	This study
pDBS222	*ampR* ORF from pDBS215 subcloned as *Kpn*I-*Sac*I upstream of and inframe with 3x V5 tag in pDBS206	This study
pDBS234	3x V5-tagged *ampR* ORF from pDBS222 subcloned as a *Kpn*I-*Not*I fragment into mini-CTX2	This study

For ChIP-Seq studies, AmpR was tagged at the carboxy-terminus with a 3x-V5 epitope tag. Briefly, the 3x-V5 epitope was polymerase chain reaction (PCR)-amplified from the plasmid ZM474 (24) using primers DBS_V5F and DBS_V5R containing *Kpn*I and *Nhe*I sites, respectively. Termination codons were included in all three reading frames with the *Nhe*I site to prevent runoff translation. The 3x-V5 amplicon was cloned into pCR2.1 TOPO (Invitrogen) to generate plasmid pDBS206 and sequenced to ensure absence of any mutations. The 1112-bp *ampR* ORF with the native promoter but without the stop codon was PCR-amplified using primers DBS_*ampR*F1 (with a *Kpn*I site) and DBS_*ampR*R (with a *Sac*I site), cloned into pCR 2.1 TOPO (pDBS215) and sequenced. The *Kpn*I-*Sac*I fragment was subcloned in-frame with the 3x-V5 tag in pDBS206 to generate plasmid pDBS222. The 3x V5-tagged *ampR* was then moved into mini-CTX2 [pDBS227; (25)] as a *Kpn*I-*Nhe*I fragment, generating plasmid pDBS234. After confirmation by PCR and restriction digestion, the suicide plasmid pDBS234 was moved into PAOΔ*ampR* by electroporation (26). This resulted in strain DBS248 with a single chromosomal copy of tagged *ampR* that was then used for the ChIP-Seq studies. Functionality of the tagged AmpR in DBS248 was verified by determining the minimum inhibitory concentration (MIC) of the β-lactams, ampicillin-sulbactam and amoxicillin, and by ChIP–quantitative real-time polymerase chain reaction (qPCR).

All strains were grown in standard LB media with aeration, unless otherwise specified. Synthetic succinate medium (SSM) was used as the iron-limited media (27) and contained (g/l) K_2_HPO_4_ 6.0, KH_2_PO_4_ 3.0, (NH_4_)_2_SO_4_ 1.0, MgSO_4_.7H_2_O 0.2, sodium succinate 4.0, pH 7.0. Antibiotics were used at the following concentrations: for *Escherichia coli*: gentamycin 15 µg/ml, tetracycline 15 µg/ml, ampicillin 100 µg/ml; for *P. aeruginosa*: gentamycin 75 µg/ml, tetracycline 60 µg/ml, carbenicillin 150 µg/ml.

### Library preparation for RNA-Seq analysis

Total RNA was isolated from PAO1 and PAOΔ*ampR* with and without sub-MIC β-lactam stress, and harvested at the same growth phase as described previously (21) using the hot-phenol extraction protocol (28). RNA quality was analyzed on the Agilent Bioanalyzer and those with RNA Integrity Numbers of 8.0 or above were used for rRNA depletion using the MICROBExpress Kit (Ambion). After rRNA depletion, cDNA synthesis was performed using the SuperScript III First-Strand Synthesis System (Invitrogen) as per manufacturer protocols. Terminal transferase (New England Biolabs) and dATP (New England Biolabs) were used to add a 100–300-base poly-A tail to the cDNA samples following manufacturer instructions. The 3′ ends were then blocked with biotinylated ddATP (Perkin Elmer) using terminal transferase (New England Biolabs), followed by cleanup with the MinElute cleanup kit (Qiagen). Tailed and blocked samples were then processed on the HeliScope Single Molecule Sequencer at the Molecular Biology Core Facility, Dana Farber Cancer Institute, Boston, MA.

### ChIP-Seq sample preparation

For ChIP-Seq studies, the 3x-V5-tagged AmpR containing strain (DBS248) was grown and exposed to sub-MIC β-lactam stress as described previously (21). Protein–DNA interactions were then cross-linked *in vivo* with formaldehyde (final concentration of 1%) at room temperature for 20 min and quenched for 15 min with 0.25 M glycine. After three washes with 1× PBS, the cells were resuspended in 1 ml lysis buffer (10 mM Tris pH 8.0, 100 mM NaCl, 1 mM EDTA, 0.5 mM EGTA, 0.1% deoxycholic acid and 0.5% N-laurylsarcosine) containing a protease inhibitor cocktail (Roche). After chilling on ice, the cells were sonicated to shear the DNA to a size range of 0.5–1 kb. Cellular debris were removed by centrifugation and a 3 -µl aliquot of the supernatant was checked on an agarose gel. A 50 -µl aliquot of the supernatant was stored as the input DNA and the rest was immunoprecipitated using DynaBeads Protein G (Life Technologies), which was previously equilibrated and bound with anti-V5 monoclonal antibody (Sigma) as per manufacturer instructions. After immunoprecipitation overnight, the beads were washed five times with RIPA buffer (50 mM HEPES pH 7.5, 500 mM LiCl, 1 mM EDTA, 1% NP40, 0.7% deoxycholic acid, 50 mM NaCl in 1× TE). The beads were resuspended in 100 µl elution buffer (50 mM Tris-HCl, 10 mM EDTA, 1% SDS) at 65°C for 30 min, centrifuged to remove residual beads and incubated at 65°C overnight to reverse the cross-link. TE buffer was then added (100 µl) and the samples were treated with RNase (37°C for 2 h). The immunoprecipitated proteins were removed with proteinase K (55°C for 2 h), and the DNA was cleaned using the Qiagen Mini Reaction Cleanup kit. RNase and proteinase K treatment was also performed for the input DNA. DNA concentrations were determined using Quant-iT PicoGreen dsDNA Kit (Life Technologies). Before proceeding further, AmpR occupancy of the *ampC* promoter was determined using qPCR as described previously (21). The DNA samples were then poly-A-tailed, blocked with biotinylated ddATP, purified and processed on the Helicos sequencer as described in the RNA-Seq section.

### Data analysis for RNA-Seq and ChIP-Seq

The raw data files for both RNA-Seq and ChIP-Seq experiments from the Helicos sequencing runs were first filtered based on read length, and converted to the FASTA format using Helisphere Open Source project. CLC Genomics Workbench, version 5 (CLC Bio), was used to map the sequence reads to the *P. aeruginosa* PAO1 genome (NCBI reference sequence NC_002516.2) and for all further data analyses.

For RNA-Seq analysis, the total number of reads per gene between samples was normalized using RPKM ([reads/kb gene]/[million reads aligning to the genome]) (29). Pair-wise comparisons were performed using the RPKM values to obtain differential gene expression data. Significance of the differentially expressed genes was determined using the chi-square test on the RPKM values. Bonferroni correction was then performed and false discovery rate *P*-values were calculated. Only genes with a proportions fold-change of ≥2.0 and with Bonferroni-corrected *P*-value of ≤0.05 were considered for further analyses.

For ChIP-Seq samples, after mapping the reads to the reference genome, the peaks were detected using a window size of 500, and were shifted sequentially based on read length. The peaks that were identified were filtered on a maximum probability of 1.0E–04 for identical locations of forward and reverse reads.

### Comparison of microarray and RNA-Seq

To get a comprehensive picture of the AmpR regulon in *P. aeruginosa*, transcriptomics studies using DNA microarrays (21) and RNA-Seq (this study) were performed. Both studies used the same two strains (PAO1 and PAOΔ*ampR*), under identical conditions (without and with sub-MIC β-lactam exposure). Both EdgeR (30) and CLC genomics were used to analyze the data. Compared with our CLC analysis, the number of genes that were identified in each of the four conditions was much lower with EdgeR (data not shown). In addition, genes of some of the phenotypes that we have confirmed previously did not show up with the EdgeR analysis. Even though EdgeR has been shown to be a better package (30), RPKM analysis worked better for our dataset. It remains to be understood why such a discrepancy exists. However, the use of RPKM for the analysis of both RNA-Seq and ChIP-Seq data is well established (31–34). Further, the CLC Genomics Workbench has RPKM in-built, the widely used software for high-throughput expression data analysis. The normalized data from the microarrays and RNA-Seq were also compared to compute the extent of correlation. The Pearson correlation coefficients were determined for the four different conditions.

### Enrichment of functional categories

The gene sets that were positively and negatively regulated by AmpR without and with β-lactam stress were functionally categorized based on the *Pseudomonas* Genome Database (35). Gene distribution under individual categories in PAO1 was considered as 100% and the relative distributions in each of the four gene sets were plotted. Enrichment of specific functional categories in the individual datasets was determined using GOEAST (36). The log odds-ratio was calculated for the GOEAST enrichment. The larger the value of the ratio, the higher the relative abundance of the GO term as compared with a random condition.

### Quantitative real-time PCR assays

Genes that were differentially regulated in the RNA-Seq analysis and those that had not been tested in our previous transcriptome studies (21) were selected for qPCR confirmation. RNA isolation and cDNA synthesis were performed as described previously (21). Ten nanograms of cDNA were used per reaction well in the qPCR assays. Expression of the test genes was normalized to *clpX* (*PA1802*).

### Hydrogen peroxide sensitivity

To determine differences in hydrogen peroxide (H_2_O_2_) sensitivities of PAO1 and PAOΔ*ampR*, gradient plate assay was used (37). Briefly, 37 ml of LB agar, held at 50°C, was supplemented with 4–8 µM H_2_O_2_ and poured onto tilted 90-mm petri dishes. After solidification, 37 ml of LB agar without H_2_O_2_ was poured onto the plates on a flat surface to generate a gradient. OD600-normalized overnight LB cultures of the strains were then used to form a 75-mm streak across the gradient using a cotton swab. H_2_O_2_ sensitivity was scored as the extent of growth into the gradient, compared with the control (on LB plates without H_2_O_2_). The assay was performed in triplicate and representative results are shown.

### Growth in iron-limited media

The differential growth abilities of the *ampR* mutant vis-à-vis the wild-type strain were determined on SSM (27). The cultures used for the growth curve assays were grown overnight in SSM from fresh LB plates. For the assay, the overnight cultures were normalized to an OD600 of 0.02 in SSM and the ODs of 200 -µl aliquots were monitored for 17 h at 37°C in 96-well flat-bottom tissue culture plates (Nunc). Conditions were made iron-replete by supplementing SSM with 100 µM FeCl_3_ (27).

### Temperature sensitivity assays

The ability of PAO1 and PAOΔ*ampR* to tolerate elevated temperatures was examined. Briefly, cells grown in LB broth to either the log (OD600 of 0.6–0.8) or stationary (OD600 of >2.0) phase at 30°C were exposed to 50°C for 1–3 h. Control aliquots were maintained at 30°C. Cell counts were determined before and after exposure by plating for colony forming units (CFUs).

### Statistical analyses

All the data from qPCR and phenotypic assays were examined for statistical significance using the unpaired two-tailed *t*-test on GraphPad analysis software (www.graphpad.com). RNA-Seq and ChIP-Seq data were analyzed for significance on CLC Genomics Workbench as described earlier.

## RESULTS

### RNA-Seq analysis expands the AmpR regulon

To study the function of AmpR in *P. aeruginosa*, a clean in-frame deletion strain (PAOΔ*ampR*) was constructed in PAO1 (21). PAO1 and PAOΔ*ampR* were used for gene expression profiling using RNA-Seq. To be able to compare results from the two studies, the experimental conditions for RNA-Seq were identical to the microarray experiments that were performed earlier (21). PAO1 and PAOΔ*ampR* were subjected to sub-MIC β-lactam stress before RNA isolation and cDNA synthesis as described in the Methods section.

Using RNA-Seq, the transcription profiles of PAO1 and PAOΔ*ampR* were compared in the presence (induced) and absence (uninduced) of sub-MIC β-lactam exposure. After data normalization between the replicates under each condition, the expression values across the entire genome for PAOΔ*ampR* were normalized to PAO1. The following nomenclature will be used to define AmpR-mediated positive and negative regulation. If mRNA expression levels are lower in PAOΔ*ampR* compared with PAO1, AmpR positively regulates those genes. Conversely, AmpR negatively regulates the genes if their expression levels are higher in PAOΔ*ampR.*

**Figure 1.**
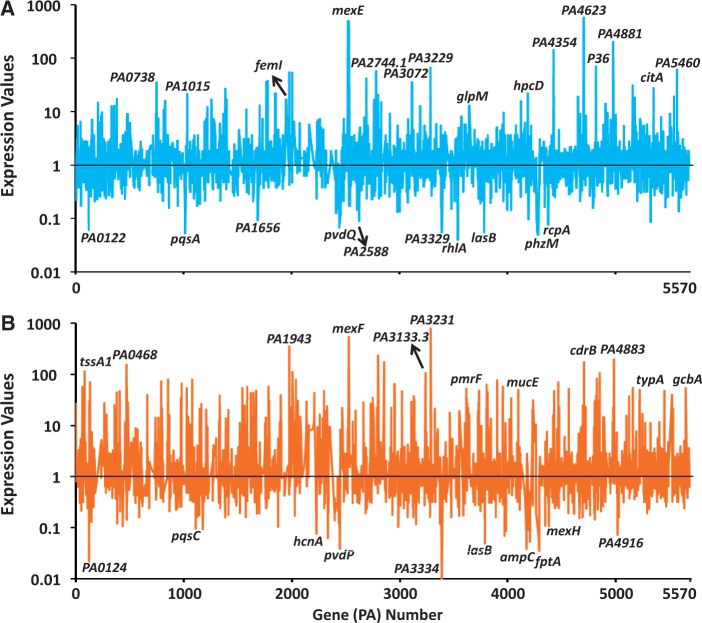
Gene expression in PAOΔ*ampR*. Relative gene expression in PAOΔ*ampR* compared with PAO1 (normalized to expression value of 1), based on RNA-Seq data, is shown in the absence (**A**) and presence (**B**) of sub-MIC β-lactam stress. Some significantly regulated genes are named. Gene annotations are from the *Pseudomonas* Genome Database (35).

The PAOΔ*ampR* data in the uninduced (Panel A, Figure 1) and induced (Panel B, Figure 1) were plotted. The expression profiles were markedly different in PAOΔ*ampR* compared with PAO1, both in the absence and presence of antibiotic exposure, attesting to the global regulatory role of AmpR in *P. aeruginosa*. As expected, the *ampC* encoding β-lactamase, which is under positive AmpR regulation (21,38), is not significantly activated in the presence β-lactam in PAOΔ*ampR* (Panel B, Figure 1). Expression of genes identified in a previous study to be positively regulated by AmpR such as the *lasB* encoding elastase (21) was also detected here (Panels A, B; Figure 1). The MexEF-OprN efflux pump that provides resistance against fluoroquinolones and chloramphenicol is negatively regulated by AmpR [Panels A, B; Figure 1, (21)]. We had demonstrated AmpR to be a positive regulator of the QS system and in agreement, key QS genes such as *pqsA* and *rhlA* are downregulated in the absence of *ampR* (Panel A, Figure 1). These findings, in addition to others discussed in the following sections, add credence to the current study.

In addition to the microarray findings, the normalized data identified two transcriptional regulators (*PA1015* and *PA2588*) and one extracytoplasmic function sigma factor (ECF) (*femI*, *PA1912*) to be differentially regulated (Panel A, Figure 1). PA2588 is a putative transcriptional regulator of the AraC family (35). Interestingly, it is located downstream of *pqsH*, which is an important component of the PQS (39). The role of AmpR in regulating the PQS is discussed in a later section. The ECF sigma factor FemI, upregulated in PAOΔ*ampR*, is part of a two-gene operon with *femR* (*PA1911*). FemR, along with FemA (PA1910), is involved in uptake of the mycobacterial siderophore mycobactin (40). Differential regulation of *femI*, along with the gene encoding the pyochelin receptor *fptA* (Panel B in Figure 1), suggests a role for AmpR in iron uptake regulation and is further explored in the later sections of this study. In addition, ncRNAs such as *P36*, *PA2744.1* and *PA3133.1* are also differentially regulated (Figure 1).

### Identification of AmpR- and AmpR-β-lactam-dependent gene sets

**Figure 2.**
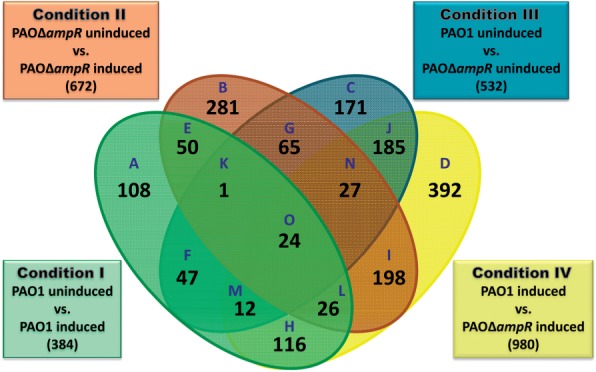
Venn diagram of differentially regulated genes. Distribution of significantly (*P* ≤ 0.01) regulated genes (≥2.0-fold) in PAO1 and PAOΔ*ampR* without (uninduced) and with (induced) sub-MIC β-lactam stress.

The RNA-Seq data were normalized and pair-wise comparisons were performed to determine differential gene expression (fold change ≥2.0, Bonferroni correction of *P* ≤0.05). The four pair-wise comparisons performed were PAO1 uninduced versus PAO1 induced (Condition I), PAOΔ*ampR* uninduced versus PAOΔ*ampR* induced (Condition II), PAO1 uninduced versus PAOΔ*ampR* uninduced (Condition III) and PAO1 induced versus PAOΔ*ampR* induced (Condition IV). This led to the identification of 2568 genes (Condition I–IV with 384, 672, 532 and 980 genes, respectively) that were differentially expressed across all four pair-wise comparisons. Although these numbers are indicative, they are not a true measure of the AmpR regulatory repertoire due to potential overlaps, i.e. individual genes could be differentially expressed under more than one condition. To address this issue, the pair-wise comparison data were plotted using a four-way Venn diagram (Figure 2). Of the 2568 genes, 865 redundant and 1703 non-redundant (Figure 2) representing 31% of the PAO1 genome were determined. Further, genes that were dependent on AmpR alone, β-lactam alone and AmpR and β-lactam were identified in accordance with the expression criteria listed in Supplementary Table S2. For example, genes in Category B (Figure 2) are regulated in an AmpR-dependent manner, independent of β-lactam exposure.

Twenty-four genes (Category O) were omitted from further analysis because they were differentially regulated under all conditions, irrespective of AmpR and/or antibiotic exposure. An additional 56 genes could not be assigned unambiguously for analysis [Category H (44 genes), Category K (1 gene), Category M (4 genes) and Category N (7 genes)] were omitted. Thus, of the remaining 1623 genes, we identified 654 AmpR-dependent (Supplementary Table S3), 483 AmpR-dependent only in the presence of β-lactam (Supplementary Table S4) and 486 differentially expressed in response to β-lactam independent of AmpR (Supplementary Table S5).

### AmpR-regulated genes are enriched in specific functional categories

**Figure 3.**
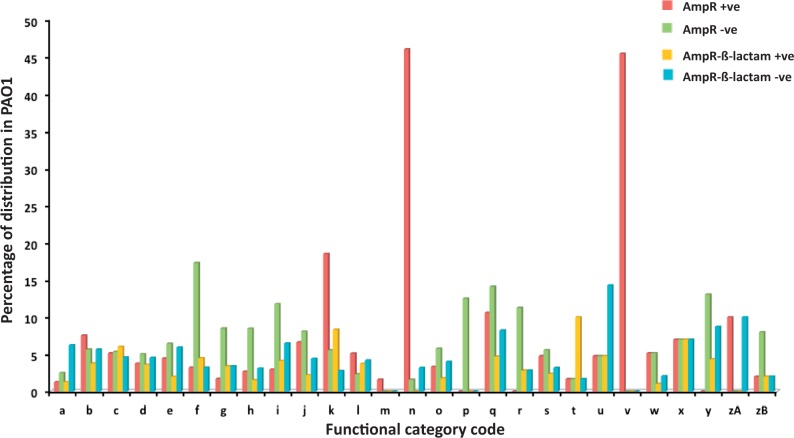
Functional categorization of AmpR-regulated genes. AmpR differentially regulated genes are functionally categorized and expressed as a percentage of the respective category in the PAO1 genome (35). The functional categories are (a) DNA replication, recombination, modification and repair; (b) fatty acid and phospholipid metabolism; (c) hypothetical; (d) membrane proteins; (e) amino acid biosynthesis, metabolism; (f) translation, post-translational modification, degradation; (g) cell wall/lipopolysaccharide/capsule; (h) transport of small molecules; (i) energy metabolism; (j) biosynthesis of cofactors, prosthetic groups, carriers; (k) adaptation, protection; (l) transcriptional regulators; (m) two-component regulatory systems; (n) secreted factors toxins, enzymes, alginate; (o) putative enzymes; (p) chaperones, heat-shock proteins; (q) central intermediary metabolism; (r) nucleotide biosynthesis and metabolism; (s) carbon compound catabolism; (t) motility and attachment; (u) chemotaxis; (v) related to phage, transposon, plasmid; (w) non-coding RNA genes; (x) protein secretion, export apparatus; (y) cell division; (zA) antibiotic resistance, susceptibility; (zB) transcription, RNA processing, degradation.

Functional categorization of the AmpR-regulated genes was as per *Pseudomonas* Genome Database annotation [(35), Figure 3]. The AmpR-regulated genes were expressed as a percentage of the distribution of each functional category in the PAO1 genome (taken as 100%). This revealed a gross upregulation of genes related to phage, transposon or plasmid (45% genes of Category ‘v’; Figure 3), as in the previous transcriptome study (21). Similarly, genes of functional class ‘n’ (secreted factors: toxins, enzymes and alginate) were also shown to be under AmpR positive regulation (46% of genes of the Category ‘n’; Figure 3). This is in agreement with previous study demonstrating that *ampR* mutant is impaired in production of extracellular enzymes such as LasA and LasB (21). AmpR also positively regulates genes belonging to functional categories ‘k’ (adaptation and protection; 19%), ‘zA’ (antibiotic resistance and susceptibility; 10%) and ‘q’ (central intermediary metabolism; 11%) (Figure 3). This finding agrees with our previous observations from DNA and phenotypic microarrays that AmpR is an important regulator of antibiotic resistance, cell wall recycling enzymes and metabolism in *P. aeruginosa* (21).

In the absence of β-lactam, AmpR negatively regulates genes involved in translation (Category ‘f’ 17%), energy metabolism (Category ‘i’ 12%), nucleotide metabolism (Category ‘r’ 11%), cell division (Category ‘y’ 13%), and chaperones and heat shock (Category ‘p’ 13%). In the presence of β-lactam stress, AmpR positively regulates genes involved in motility and attachment (Category ‘t’ 10%) and negatively regulates genes involved in chemotaxis (Category ‘u’ 15%; Figure 3).

To determine whether the functional categorization in the different gene sets is significant, enrichment analysis was performed using GOEAST (36). Primarily, gene ontology identifications (GOIDs) belonging to biological processes and cellular components were enriched in the AmpR positively and negatively regulated gene sets, respectively (Supplementary Table S6). GOIDs belonging to ribosomal protein biosynthesis and oxidative phosphorylation were statistically significantly enriched in the AmpR-dependent negatively regulated gene set (log odd ratio >1.5; *P* < 0.05; Supplementary Table S6). There was no enrichment in the AmpR-β-lactam-dependent gene sets.

In the AmpR positively regulated set, many genes involved in pyoverdine biosynthesis were significantly enriched (log odd ratio >2.5, *P* < 0.03; Supplementary Table S6). This enrichment is, in part, due to the presence of genes encoding the major catalase KatA, and the superoxide dismutase SodA. The physiological effects of enrichment of these genes on iron acquisition and oxidative stress response, and the role of AmpR in their regulation, are discussed in later sections. Furthermore, GOIDs containing genes such as the QS regulator *rhlR* and the stress-phase sigma factor *rpoS*, which were identified to be AmpR-regulated previously (21), also showed significant enrichment (Supplementary Table S6).

### Regulation of small RNAs by AmpR

RNA-Seq allows detection of expression profiles of small RNAs (sRNAs). Previous microarray studies with PAOΔ*ampR* showed dysregulation of the small regulatory RNA rgRsmZ (21). This led us to hypothesize that other sRNAs may also be AmpR-regulated but were not detected in the microarray studies due to technical limitations. As hypothesized, RNA-Seq analysis of the *ampR* mutant identified many ncRNAs, both in the absence and presence of sub-MIC β-lactam exposure (Supplementary Tables S3 and S4). Some of these were tRNAs, which is expected given their abundance in the cell. Downregulated ncRNAs in PAOΔ*ampR* (AmpR positive regulation) include *P8* (*PA1030.1*; uninduced – 30-fold, *P* 2.14E-06; induced – 92-fold, *P* 1.03E-14) and *prrF1* (*PA4704.1*; uninduced – 4-fold, *P* 0.00E+00; induced NS), whereas expression of *P7* (*PA0887.1*; uninduced 6.3-fold, *P* 0.00E+00; induced 3.1-fold, *P* 5.35E-14) and *amiL* (*PA3366.1*; uninduced 2.5-fold, *P* 1.82E-13; induced NS) was upregulated (AmpR negative regulation). Interestingly, rgRsmZ, which was detected in the microarray analysis, was not detected in RNA-Seq but dysregulation was confirmed by qPCR (discussed in the following sections).

AmpR-mediated regulation of some of the sRNAs was determined by qPCR. AmpR was found to positively regulate expression of *P34* [*PA5181.1*; Relative Quantity (RQ): uninduced 0.39 ± 0.06, *P* 0.0003; induced 0.38 ± 0.016, *P* 0.002]. Expression of *P34* requires RpoS (41). Because AmpR positively regulates the expression of *rpoS* (21), AmpR regulation of *P34* is likely via RpoS. Positive regulation of *P32* (*PA4758.1*) by AmpR and its physiological effects on the *ampR* mutant strain are discussed in the section on heat-shock response. Further, AmpR was also found to positively regulate the antisense RNA asPrrF1 (Supplementary Table S3) and is discussed further in the following section.

### AmpR regulates iron uptake positively

Iron is critical in many biological reactions across kingdoms, and *P. aeruginosa* is no exception. However, freely available iron is in a poorly soluble and biologically unusable ferric (Fe^3+^) form at neutral pH in aerobic conditions (42). To circumvent this issue, *P. aeruginosa* has evolved high-affinity iron uptake systems mediated by siderophores. Siderophores are iron chelators that bind extracellular iron and transport it to receptors on the cell surface [reviewed in (42)]. *Pseudomonas aeruginosa* can produce and take up heme– or iron–siderophore complexes (43). In addition, *P. aeruginosa* also synthesizes outer membrane receptors for siderophores produced by other bacteria, including pyoverdines produced by other pseudomonads (44), and aerobactin (45) and enterobactin (45,46) produced by Enterobacteriaceae. *Pseudomonas aeruginosa* produces two main types of siderophores, pyoverdine and pyochelin (42). Pyoverdine is the green-yellow fluorescent pigment that is produced typically under conditions of iron limitation (47). The *pvd* genes involved in the synthesis of pyoverdine are clustered (*PA2385*–*PA2426*) on the PAO1 genome (35). RNA-Seq analysis of the *ampR* mutant revealed downregulation (3- to 103-fold) of many *pvd* genes (Supplementary Table S3). This includes *pvdS* (*PA2426*), the ECF sigma factor (uninduced – 7.6, *P* 3.49E-09; induced – 38.2, *P* 1.93E-08) that is known to regulate expression of the *pvd* genes (48). Genes encoding the second siderophore system, pyochelin, are part of a gene cluster (*PA4220*–*PA4231*) and consist of three operons (*PA4220–PA4221*, *PA4222–PA4226* and *PA4228–PA4231*). Genes of these operons (*PA4224*–*PA4226*, *PA4228*–*PA4231*) are significantly downregulated (14-fold to 193-fold) in PAOΔ*ampR* in a β-lactam-independent manner (Supplementary Table S3). This is also reflected in the GOEAST analysis of the AmpR positively regulated gene set revealing a significant enrichment (log odds ratio ≥1.0, *P* ≤ 0.05) of the *pvd* genes (Supplementary Table S6). These findings suggest that AmpR is potentially involved in iron uptake.

**Figure 4.**
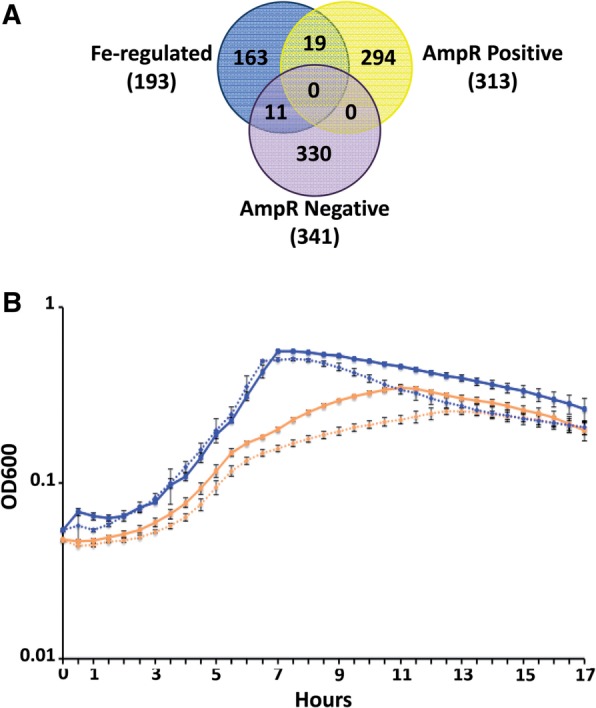
AmpR regulates iron uptake. (**A**) Comparing the AmpR positively and negatively regulated genes against the iron-regulated gene set shows overlaps between the datasets. (**B**) Growth in iron-limited media of PAO1 (solid lines) and PAOΔ*ampR* (dashed lines) in the absence (orange lines) and presence (blue lines) of exogenously added FeCl_3_.

Further, comparison of AmpR-regulated genes from this study with the iron-related genes identified as part of a previous transcriptome meta-analysis (49) revealed overlaps (Figure 4A). Genes involved in pyochelin biosynthesis are part of the 19 that are shared between the iron-regulated and AmpR positively regulated datasets (Figure 4A). The overlapping set also includes *prpL* and *pfeR* encoding a PvdS-regulated protease and a transcriptional regulator, respectively. PrpL has been implicated in virulence (50). PfeR, a two-component response regulator, positively regulates *pfeA,* encoding the enterobactin outer membrane receptor (51). Moreover, positive regulator of iron uptake, asPrrF1 is also downregulated in PAOΔ*ampR* in the RNA-Seq (uninduced: 4-fold, *P* < 0.001; induced: NS) as well as qPCR (uninduced: 0.47 ± 0.04, *P* 0.004; induced: NS) assays. However, expression of the master repressor of iron uptake Fur (52) is not significantly differentially regulated in the *ampR* RNA-Seq analyses. Downregulation of genes involved in siderophore biosynthesis, and comparison with previous meta-analysis led us to hypothesize that AmpR plays a positive regulatory role in iron uptake.

To confirm the role of AmpR in iron uptake, growth curves were performed in iron-limited SSM (27). Loss of *ampR* resulted in impaired growth in SSM compared with PAO1 (orange lines; Figure 4B). Maximum growth difference between these strains was seen in the log and early stationary phases (*P* < 0.0001 at all time points between 4 and 14 h) of growth (orange lines; Figure 4B). The reduced growth of PAOΔ*ampR* in iron-deficient media can be a result of impairment in either uptake or utilization of iron. To address this, growth curves were performed with exogenously added FeCl_3_, making the media iron-replete (blue lines; Figure 4B). Addition of iron to the media enhanced the growth rate of both PAO1 and PAOΔ*ampR* (blue lines; Figure 4B), compared with growth in SSM alone (orange lines; Figure 4B). Moreover, under iron-replete conditions, growth of the two strains is very similar till about 10 h, after which the *ampR* mutant shows significantly accelerated death (blue lines; Figure 4B). Thus, in the presence of excess iron in the log phase, PAOΔ*ampR* shows no growth deficiency. This observation, and the transcriptome data, strongly suggests that AmpR plays a role in iron uptake, and not in iron utilization. The accelerated death phase seen under iron-replete conditions with PAOΔ*ampR* between 10 and 16 h is significant (blue lines, Figure 4B; *P* ≤ 0.0003 at all points). This is possibly due to the fact that PAOΔ*ampR* uses up all the freely available iron to maintain growth rates similar to PAO1 for the first 10 h of the experiment. When conditions start to become iron limiting (after 10 h, Figure 4B), continued growth of PAOΔ*ampR* is hampered due to impaired iron uptake. Moreover, under iron-replete conditions, neither strain produced pyoverdine, seen visually as a lack of yellow-green color of the cultures (data not shown), suggesting pyoverdine-independent iron uptake. *Pseudomonas aeruginosa* also has an uncharacterized low-affinity iron uptake system that functions under iron-replete conditions (P. Cornelis, personal communication) and a citrate-mediated iron uptake system (53), potentially explaining growth.

In addition to the siderophore-mediated uptake, expression of the gene encoding heme acquisition protein HasAp (PA3407) is downregulated in an AmpR-β-lactam-dependent manner (34.9-fold, *P* 8.5E-08; Supplementary Table S4). This further attests to the role of AmpR in iron uptake in *P. aeruginosa*. The pH of the media is known to influence growth in SSM (27), but there was no difference in the pH of the media between the strains (data not shown). Thus, the gene expression and phenotypic data clearly indicate a positive regulatory role for AmpR in iron uptake in *P. aeruginosa*.

### *Pseudomonas aeruginosa* AmpR regulates heat-shock response by modulating rgP32 expression

**Figure 5.**
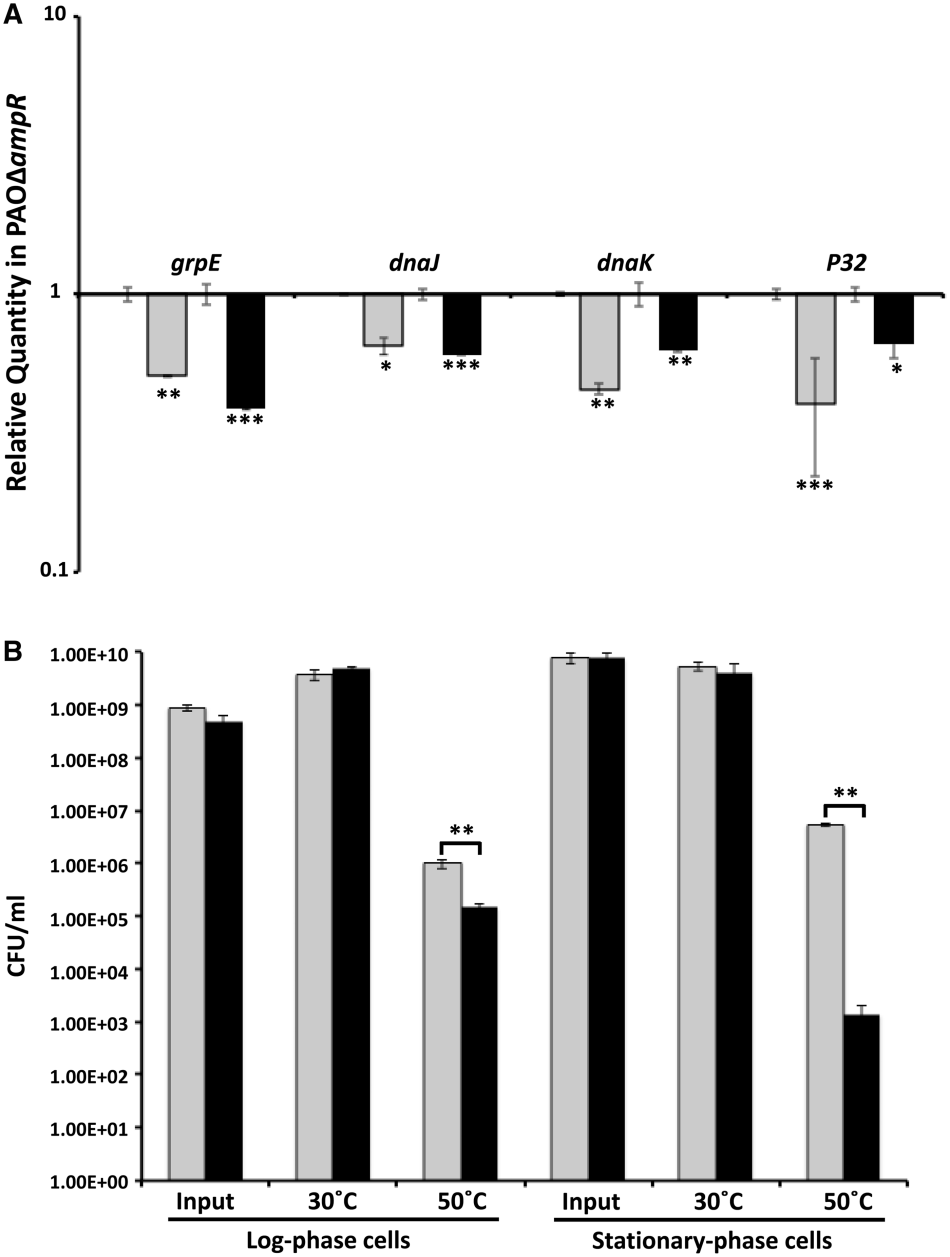
Regulation of heat-shock response by AmpR. (**A**) qPCR of *hsp70* genes: RNA was isolated from PAO1 and PAOΔ*ampR* cells, without and with sub-MIC β-lactam stress, reverse transcribed to cDNA and tested by qPCR with gene-specific primers, as described in the text. Relative gene expression in PAOΔ*ampR* is shown without (gray bars) and with (black bars) sub-MIC β-lactam exposure. Values have been normalized to expression in PAO1 under the same conditions (log10 RQ = 1) and bars above and below the threshold represent up- and downregulation, respectively. (**B**) CFU counts of heat-shock exposed and unexposed cells in the log and stationary growth phases: Cells grown at 3°C were OD600-normalized, split into two aliquots and maintained at 30° and 50°C for varying periods (3 h for log phase, 1 h for stationary phase) before enumeration. Data of PAO1 (gray bars) and PAOΔ*ampR* (black bars) are represented. *P < 0.02, **P < 0.003, ***P < 0.0005.

RNA-Seq analysis revealed that AmpR positively regulates the sRNA rgP32 (20.8-fold downregulated in PAOΔ*ampR*, *P* 7.77E-14; Supplementary Table S3). *P32* is the last gene of a three-gene operon with *dnaJ* and *dapB* (35). DnaJ is part of the Hsp70 heat-shock response system (54). The DnaJ–DnaK–GrpE (PA4760–PA4762) chaperone prevents premature folding of nascent polypeptides and, along with the GroEL (Hsp60) system, helps in the heat-shock response in bacteria (54–56). DnaK is the *P. aeruginosa* homolog of *E. coli* Hsp70 (35). Conversion between the ATP- or ADP-bound forms of DnaK is controlled by DnaJ and GrpE, which function as a co-chaperone and a nucleotide exchange factor, respectively (57). Given that the sRNA is part of the operon encoding genes involved in the Hsp70 system, we hypothesized differential regulation of the Hsp70 heat-shock system in PAOΔ*ampR* compared with PAO1. qPCR analysis showed that the expression of *grpE* (RQ: uninduced 0.51 ± 0.004, *P* 0.0017; induced 0.38 ± 0.003, *P* 0.0003), *dnaJ* (RQ: uninduced 0.65 ± 0.045, *P* 0.015; induced 0.6 ± 0.005, *P* 0.0004) and *dnaK* (RQ: uninduced 0.45 ± 0.02, *P* 0.0019; induced 0.62 ± 0.007, *P* 0.0028) is downregulated in PAOΔ*ampR* (Figure 5A). The positive regulation is independent of sub-MIC β-lactam in the system (Figure 5A).

Downregulation of the Hsp70 heat-shock system genes in the *ampR* mutant led us to hypothesize that, compared with PAO1, PAOΔ*ampR* would behave differently at higher temperatures. However, no difference in growth pattern was observed between PAO1 and PAOΔ*ampR* at 30°C, 37°C or 43°C (data not shown). The heat tolerance of the two strains was then examined by enumerating CFUs after exposure of both log- and stationary-phase cells to 50°C, inducing the cellular heat-shock response. There was no significant difference in CFU for either log- or stationary-phase cells grown at 30°C (Figure 5B). However, PAOΔ*ampR* log-phase cells were more sensitive compared with PAO1, with >90% of cells killed after 3 h of 50°C exposure (*P* 0.0018; Figure 5B). The stationary-phase PAOΔ*ampR* cells also followed a similar trend. After a one hour exposure, loss of *ampR* led to a 99.9% drop in cell viability compared with PAO1, when stationary-phase cells were exposed to 50°C (*P* 0.0014; Figure 5B).

Compared with the log phase, the stationary-phase cells were more sensitive to the elevated temperature and show a 2-log greater drop in CFU even after a brief exposure (Figure 5B). This is counterintuitive, as at stationary phase, the cells are thought to be more resistant to changing conditions as compared with log phase. Downregulation of *rpoS* encoding the stationary-phase sigma factor in PAOΔ*ampR* (21) potentially plays a role in the enhanced sensitivity to temperature. Regulation of *rpoH,* encoding the heat-shock sigma factor, was also not significantly different between the strains in the RNA-Seq analysis (data not shown). This, however, is not surprising because expression of many sigma factors, including RpoH, is regulated post-transcriptionally (58,59). Dysregulation of the *hsp70* genes, confirmed by reduced temperature tolerance of PAOΔ*ampR*, suggests a positive regulatory role for AmpR in the heat-shock response of *P. aeruginosa*.

### AmpR positively regulates *P. aeruginosa* oxidative stress response

H_2_O_2_ is a byproduct of O_2_ metabolism whose deleterious effects on cells include altered membrane potential (60) and DNA mutation caused by single-stranded nicks (61). Intracellular H_2_O_2_ detoxification is achieved by the enzyme catalase and *P. aeruginosa* has four homologs: KatA (PA4236), KatB (PA4613), KatE (PA2147) and KatN [PA2185; (62)]. Of these, KatA is the major catalase and is expressed in all stages of cell growth but is produced more in the stationary phase (62). RNA-Seq analysis of PAOΔ*ampR* revealed downregulation of *katA* expression (−2.1-fold, *P* 1.6E-09) compared with PAO1 in the absence of antibiotic exposure, suggesting AmpR-dependent expression (Supplementary Table S3). Differential expression of *katA* was validated using qPCR (RQ uninduced: 0.12 ± 0.01, *P* 0.0012). The sRNA rgRgsA (*PA2958.1*), which requires GacA and RpoS for its expression, contributes to H_2_O_2_ resistance (63). The expression of rgRgsA is also downregulated >2-fold in PAOΔ*ampR* (Supplementary Table S3), indicating positive AmpR regulation.

**Figure 6.**
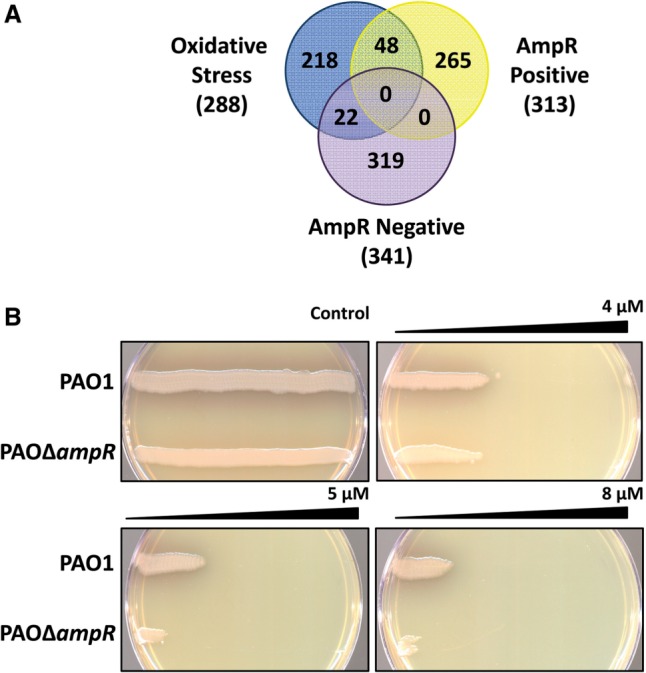
AmpR regulates resistance to oxidative stress. (**A**) Comparing the AmpR positively and negatively regulated genes against the oxidative stress gene set shows overlaps between the datasets. Overlapping genes include those identified previously to play a role in the oxidative stress response. (**B**) Gradient plates demonstrate decreased resistance of PAOΔ*ampR* to H_2_O_2_, compared with the control plate without H_2_O_2_. Representative data from four independent experiments are shown.

Previous meta-analysis studies of *P. aeruginosa* transcriptomes led to the identification of genes that were specifically differentially regulated under oxidative stress conditions (49). The differential regulation of *katA* and rgRgsA in PAOΔ*ampR* prompted comparison of the AmpR-regulated genes with the oxidative stress gene set. Seventy genes were shared between the two conditions, 48 and 22 of which are positively and negatively regulated by AmpR, respectively (Figure 6A). The 48 positively regulated genes include the major *P. aeruginosa* catalase *katA*, and four genes involved in PQS signal biogenesis [*pqsA* (*PA0996*), *pqsE* (*PA1000*), *phnA* (*PA1001*) and *phnB* (*PA1002*)]. Interestingly, 22 of the 48 genes are clustered in a single locus on the genome that is involved in the production of R- and F-type pyocins (64,65), and are located in regions of genome plasticity (RGPs), RGP03 and RGP04 (66). These genes were also identified in a previous transcriptome study to be AmpR-regulated (21). Most of these 22 AmpR-downregulated genes that are shared with the oxidative stress gene set are involved in metabolism, including six of the *nuo* genes, which synthesize components of nicotinamide adenine dinucleotide dehydrogenase I (67). Another member of the LTTR family of transcriptional regulators, OxyR regulates *katA* expression in response to oxidative stress (68). Although *ampR* deletion in PAO1 did not affect *oxyR* expression in the RNA-Seq analysis (data not shown), qPCR analysis revealed that AmpR positively regulates *oxyR* expression (RQ uninduced: 0.31 ± 0.012, *P* 0.001).

To determine whether reduced expression of *katA* and other oxidative stress-response genes translates into an observable phenotype, the H_2_O_2_ sensitivity of PAO1 and PAOΔ*ampR* was compared using the gradient plate method (37). PAOΔ*ampR* demonstrates a concentration-dependent reduced growth compared with PAO1 on the H_2_O_2_ gradient (Figure 6B), suggesting an impaired resistance to oxidative stress. This finding is in agreement with downregulation of oxidative stress response genes in PAOΔ*ampR*.

Thus, the transcriptomic and phenotypic data demonstrate a role for AmpR in positively regulating oxidative stress response in *P. aeruginosa*.

### AmpR regulates phenazine production by modulating expression of *phzA1-G1* and *phzA2-G2* operons

**Figure 7.**
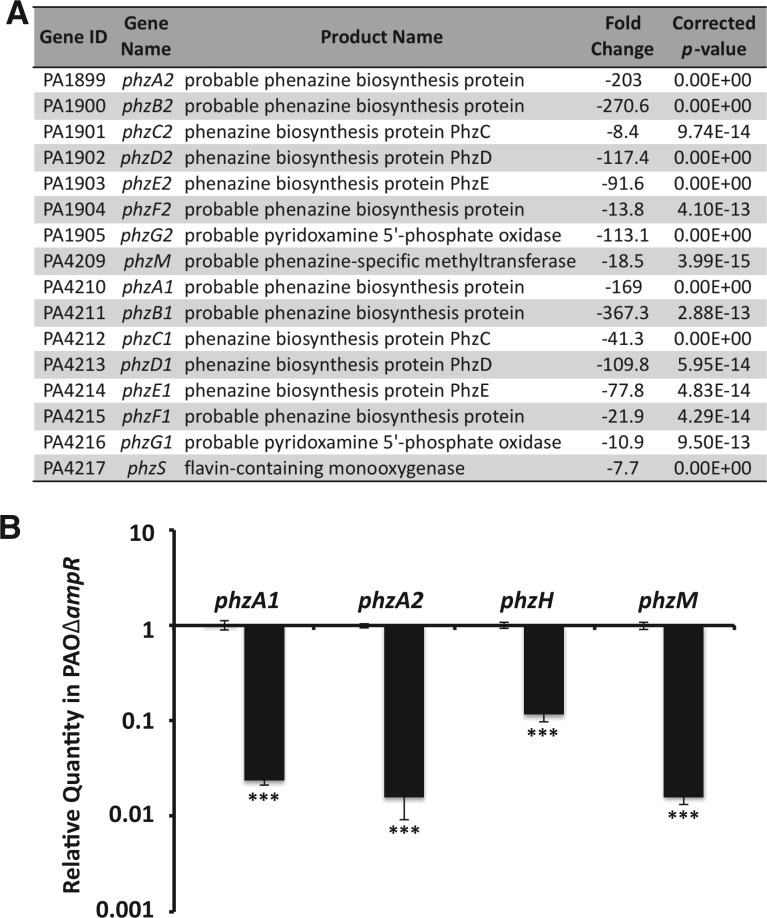
Regulation of phenazine genes by AmpR. (**A**) Genes involved in phenazine biosynthesis are significantly downregulated in PAOΔ*ampR* as seen in RNA-Seq analysis. (**B**) Differential regulation of the first genes of the phenazine biosynthetic operons *phzA1* and *phzA2*, and the modifying enzymes *phzH* and *phzM*, was validated by qPCR.***P < 0.0001.

The PAOΔ*ampR* strain is impaired in producing pyocyanin (21). *Pseudomonas aeruginosa* PAO1 and PA14 have two redundant operons *phzA1-G1* (*PA4210*–*PA4216*; *phz1* operon) and *phzA2-G2* (*PA1899*–*PA1905*; *phz2* operon) that are involved in biosynthesis of the phenazine precursor, phenazine-1-carboxylic acid (35). This precursor is then sequentially modified by a methyltransferase (PhzM, PA4209) and a monooxygenase (PhzS, PA4217) to form pyocyanin (69). The expression of all the genes involved in pyocyanin biosynthesis and export (*phz1* and *phz2* operons, *phzM*, *phzS*, *mexGHI-opmD*) was significantly reduced in the *ampR* mutant (positive AmpR regulation) in the RNA-Seq analysis (Figure 7A and Supplementary Table S3). The positive AmpR regulation of *phzA1*, *phzA2* (the first genes of *phz* operons), *phzH* and *phzM* was also confirmed by qPCR using gene-specific primers (Figure 7B). The GOIDs under which *phzM* and *phzS* are classified (antibiotic metabolic processes, antibiotic biosynthetic processes and drug metabolic processes) also showed functional enrichment (log-odd ratio 3.46, *P* 0.03; Supplementary Table S6).

The *phz1* operon is under QS control, whereas *phz2* operon regulators have not been identified (69,70). AmpR-mediated positive regulation of the *phz2* operon is a novel finding.

### The PQS is positively regulated by AmpR

**Figure 8.**
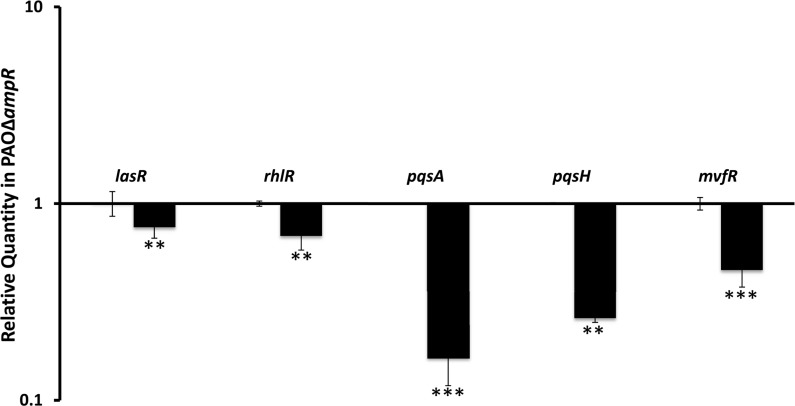
AmpR positively regulates critical QS regulators of the Las system, Rhl system and PQS. Expression of the genes was determined by qPCR. The expression levels in PAOΔ*ampR* are shown, normalized to expression in PAO1. *P < 0.02, **P < 0.003, ***P < 0.0005.

Of the 654 AmpR-dependent genes, 313 and 341 are positively and negatively regulated, respectively. Genes that are positively regulated by AmpR include the QS-controlled genes *lasA*, *lasB*, *rhlAB*, *rhlR* and *hcnABC* operon (Supplementary Table S3). The positive regulation of *lasR* and *rhlR* expression by AmpR was confirmed by qPCR (Figure 8). Furthermore, even under sub-MIC β-lactam exposure, AmpR positively regulated *lasR* (RQ induced: 0.84 ± 0.03, *P* 0.001) and *rhlR* (RQ induced: 0.71 ± 0.007, *P* 0.0001) expression. These concur with our previous findings that AmpR is a positive regulator of some QS phenotypes (21).

The PQS is a critical part of QS signaling in *P. aeruginosa*, and complements the *las* and *rhl* systems (71,72). Genes of the two operons, *pqsABCDE* (*PA0996–PA1000*) and *phnAB* (*PA1001–PA1002*), and *pqsH* (*PA2587*) are involved in PQS biosynthesis (73). PhnAB converts the PQS precursor chorismate to anthralinate, which is further converted to the signaling molecule PQS by PqsA-D and PqsH (39,73). The RNA-Seq data show that AmpR positively regulates all these genes except for *pqsH* (Supplementary Table S3). We validated the RNA-Seq data using qPCR of the first gene of the *pqs* operon (*pqsA*) and *pqsH* (Figure 8). MvfR (PA1003) lies downstream of the *pqs* genes and positively regulates genes in this cluster (74,75). qPCR analysis revealed that AmpR positively regulates *mvfR* expression (Figure 8). Thus, it is likely that AmpR-mediated regulation of the PQS in *P. aeruginosa* is via MvfR.

*Pseudomonas aeruginosa qscR* (*PA1898)* encoding another QS regulator is located in the same locus as the *phzA2* operon (76). qPCR analysis showed that the expression of *qscR* is significantly reduced in PAOΔ*ampR* (RQ: uninduced– 0.38 ± 0.17, *P* 0.0057). This further supports the role of AmpR as a QS regulator.

### V5-tagged AmpR is functional *in vivo*

The previous study and current study suggest that the AmpR regulon in *P. aeruginosa* is extensive (21,22). However, it is highly unlikely that all the genes are under direct AmpR regulation. It is possible that AmpR indirectly controls a subset of genes via other regulators. Accordingly, we identified transcriptional regulators that could be potential targets of AmpR [Supplementary Tables S3 and S4, (21)]. Moreover, using the putative binding site (77), *in silico* analysis of the *P. aeruginosa* PAO1 genome identified genes that may be direct targets of AmpR (21). Some of these targets were confirmed to be differentially regulated using DNA microarrays (21). However, direct AmpR targets have not been demonstrated as yet.

To identify the direct targets of AmpR, ChIP-Seq studies were performed using a 3x-V5-tagged AmpR. As AmpR has a positive regulatory role in β-lactam resistance, the functionality of the tagged AmpR was verified by determining the MIC. Amoxicillin had an MIC of 4 µg/ml for PAOΔ*ampR*, whereas the wild-type PAO1 is resistant (>256 µg/ml). The MIC of amoxicillin on PAOΔ*ampR*::*ampR*-V5 was >256 µg/ml, similar to PAO1, indicating that C-terminus tagging did not inhibit AmpR function. ChIP was then performed both in the presence and absence of sub-MIC β-lactam exposure. Before proceeding with the high-throughput sequencing, validity of the pull-down assay was tested by qPCR for P*_ampC_*. AmpR occupancy data for the *ampC* promoter revealed that compared with the input DNA, the ChIP DNA showed a 17.1 ± 1.2-fold and 21.5 ± 4.3-fold higher occupancy in the absence and presence of β-lactam stress, respectively. This demonstrated that the tagged AmpR was able to effectively bind the *ampC* promoter *in vivo*. Thus, the MIC and ChIP–qPCR studies confirmed *in vivo* functionality of the V5-tagged AmpR protein.

### Identifying direct AmpR targets by ChIP-Seq

After confirming functionality of the tagged AmpR protein *in vivo*, ChIP and input DNA samples were processed on the Helicos sequencer. Data analysis was performed on the CLC Genomics Workbench as described in the Methods section. The target regions on the PAO1 genome that AmpR binds are shown in Table 2. All the loci in the table have significant Wilcoxon *P* < 3.0E-05. In agreement with the ChIP–qPCR data (previous section), ChIP-Seq data showed that AmpR binds to promoter DNA upstream of *ampC* under both induced and uninduced conditions (Table 2). This is typical of LTTRs, which bind their target sequences irrespective of effector binding (78,79), and is also seen in the P*_ampC_* ChIP–qPCR (previous section).

**Table 2.** AmpR ChIP peaks

Chromosomal locus	Average read length	% Reads mapping to locus	Strand	FDR (%)	Flanking genes		FC in RNA-Seq (AmpR Regulation)
					5′	3′	
Uninduced							
586888–586930	42	88	+	2.0E–01	*rsmY*	*PA0528*	ND
797294–797374	80	53	+	7.5E–01	*PA0728*	*PA0729*	ND
901840–901853	13	95	–	1.9E–01	*PA0826*	*ssrA*	ND
1668963–1669003	40	95	+	1.0E+00	*ffs*	*PA1531*	ND
1921430–1921543	113	95	+	1.2E+00	*oprF*	*cobA*	ND
3123393–3123411	18	83	–	5.3E–02	*PA2763*	*PA2764*	ND
4057616–4057641	25	89	–	1.6E–09	*fdxA*	*rsmZ*	2.0 (–ve)
4592895–4593005	110	97	–	1.3E–04	*PA4108*	*ampR*	ND
4362457–4362649	192	88	+	1.6E+00	*PA4140*	*PA4141*	ND
4782725–4783036	311	89	–	3.4E+00	*rplA*	*rplK*	2.1 (–ve)
4956459–4956671	212	91	–	1.9E+00	*rnpB*	*PA4422*	ND
5387789–5387840	51	95	–	6.8E–02	*PA4802*	*PA4802.1*	ND
5884393-5884467	74	94	+	1.3E+00	*ssrS*	*PA5228*	ND
5986032–5986133	101	90	–	1.7E+00	*rpmG*	*rpmB*	2.2 (+ve)
6183549–6183590	41	88	–	2.6E+00	*PA5492*	*polA*	ND
Induced							
586884–586929	45	96	+	2.3E–03	*rsmY*	*PA0528*	ND
901794–901852	58	92	–	3.1E–03	*PA0826*	*ssrA*	ND
1668962–1669008	46	98	+	6.3E–02	*ffs*	*PA1531*	41.3 (+ve)
3206872–3207029	157	96	+	1.0E–02	*PA2852.1*	*oprI*	ND
4057616–4057641	25	88	–	3.2E–05	*fdxA*	*rsmZ*	ND
4592896–4593007	111	73	–	2.5E–03	*PA4108*	*ampR*	ND
4956491–4956668	177	91	–	1.9E–03	*rnpB*	*PA4422*	ND
5308608–5308852	244	93	+	3.8E–02	*crcZ*	*PA4726.2*	ND
5387787–5387840	53	94	–	7.2E-02	*PA4802*	*PA4802.1*	ND
5884382–5884467	85	93	+	3.7E–01	*ssrS*	*PA5228*	ND
6183549–6183593	44	90	–	1.3E+00	*PA5492*	*polA*	2.4 (–ve)

ChIP-Seq studies were performed on *P. aeruginosa* PAOΔ*ampR* strain harboring V5-tagged AmpR in the absence (uninduced) and presence (induced) of sub-MIC β-lactam stress. Regions on the chromosome that were enriched in the ChIP DNA samples compared with the input DNA are shown. All readings have a Wilcoxon Filter *P* ≤ 3.0E-05. FDR, False discovery rate; FC, Fold change; ND, Not detected.

The region with the least % false discovery rate value, irrespective of inducer presence, is within rgRsmZ (*PA3621.1*). The locus that was pulled down in ChIP is on the negative strand (Table 2) and corresponds to a 25-bp region in the *rsmZ* gene. AmpR-dependent regulation of the *rsmZ* gene was also seen previously in transcriptome studies using microarrays (21) and in the current RNA-Seq analysis (Supplementary Table S3). The rgRNAs *rsmY* and *rsmZ* are thought to be functionally redundant (80) and play a major role in the acute to chronic lifestyle transition of *P. aeruginosa* (81–83). Transcription of *rsmY* and *rsmZ* is repressed by NarL [PA3879, (84)], CafA [PA4477, (85)] and MvaT/U [PA4315, (83)], and is activated by GacA [PA2586, (86,87)].

**Figure 9.**
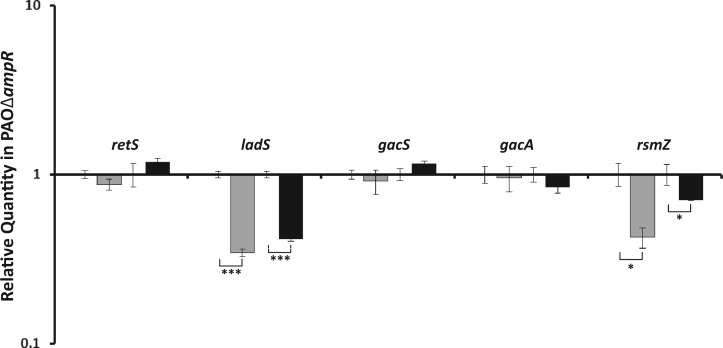
Quantitative PCR analysis of Gac-Rsm pathway genes. Relative expression of genes of the RetS-LadS-GacSA-Rsm pathway in PAOΔ*ampR* compared with PAO1 was analyzed in the absence (gray bars) and presence (black bars) of sub-MIC β-lactam stress. Gene expression in the *ampR* mutant has been normalized to the corresponding condition in the wild-type strain and expressed as relative numbers of gene-specific transcripts. *P ≤ 0.02, ***P < 0.0001.

In an attempt to understand the role of AmpR in this process, qPCR assays were performed with the other players of this well-established regulatory cascade (Figure 9). Two hybrid sensor kinases LadS (PA3974) and RetS (PA4856) have a positive and negative effect, respectively, on the sensor kinase GacS (81,82). GacS activates transcription of rgRsmZ and rgRsmY indirectly through GacA (83). The rgRNAs *rsmY* and *rsmZ* sequester the RNA-binding protein RsmA (PA0905), which plays a key role in regulating > 500 genes, controlling the acute versus chronic lifestyle transition (88). qPCR analysis revealed that in the *ampR* mutant, expression of *ladS* (RQ: uninduced 0.35 ± 0.018, *P* < 0.0001; induced 0.42 ± 0.012, *P* < 0.0001) and rgRsmZ (RQ: uninduced 0.43 ± 0.059, *P* = 0.01; induced 0.71 ± 0.006, *P* = 0.02) was reduced, indicating that AmpR is required for their expression (Figure 9). No dysregulation of *retS*, *gacS* or *gacA* was observed (Figure 9). Our previous microarray studies (21) and our current RNA-Seq, qPCR and ChIP-Seq studies show that AmpR activates transcription of *rsmZ*. Furthermore, *P. aeruginosa* AmpR is a positive regulator of acute virulence factors, many of which are QS-regulated, while negatively regulating chronic infection phenotypes such as biofilm formation (21). The current ChIP-Seq data seem to suggest that this regulation by AmpR is mediated by the regulatory RNA rgRsmZ.

### AmpR-binding site analysis

**Figure 10.**
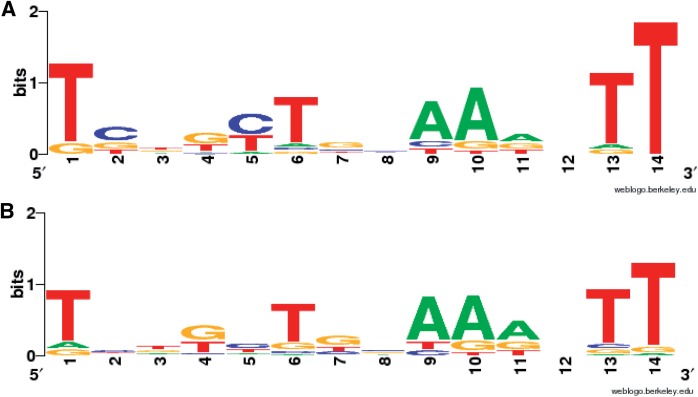
ChIP-Seq data-based AmpR-binding site analysis. Promoters of the genes that were identified by ChIP-Seq to be AmpR-regulated, either in the absence or presence of β-lactam stress, were scanned for the presence of the putative AmpR-binding motif using RSAT. The binding sites upstream of each gene were then used as input to generate a WebLogo for the uninduced (**A**) and induced (**B**). *P* 0.001.

The ChIP data were used to identify the AmpR-binding site. A region of about 200 bp upstream of the genes that were identified to be AmpR-bound (Table 2) was used as input for regulatory sequence analysis tools [RSAT; rsat.ulb.ac.be; (89)]. These regions were queried using the putative AmpR-binding site identified earlier (77) and the matrix derived as part of genome-wide analysis (21). Using the binding sites identified in the promoter regions of the AmpR-regulated genes as input for RSAT, WebLogos were generated for both uninduced and induced datasets (Figure 10). The DNA motif that AmpR seems to bind, both in the presence and absence of effectors, is almost identical, except for minor changes at positions 1, 2, 3 and 5 (Panels A and B, Figure 10). The AmpR motif, like the LTTR box (79), is AT-rich and the conserved consensus binding sequence is As and Ts at positions 1, 6, 9, 10, 13 and 14 (Figure 10).

### LasR is a direct target of AmpR

The regulator LasR is at the top of the QS regulatory hierarchy in *P. aeruginosa* (90–92). Other regulators, in addition to LasR, regulate the Rhl system and PQS [reviewed in (11)]. Our findings have identified that AmpR is one of these regulators that positively regulates the Las and Rhl systems transcriptionally (PQS section earlier) and phenotypically [Figure 8; (21)]. ChIP-Seq data suggested that AmpR regulates the QS master regulator LasR directly (Table 2). This finding is further strengthened by the presence of a putative AmpR-binding motif upstream of the *lasR* gene. To validate the ChIP-Seq data, ChIP–qPCR was performed for selected targets using the V5-tagged AmpR strain.

As expected, the *ampC* promoter showed 79-fold enrichment in the ChIP DNA compared with the control DNA, demonstrating strong AmpR binding. AmpR pull-down of P*_ampC_* was also confirmed using a VSVG-tagged AmpR (data not shown). ChIP–qPCR data showed that the *lasR* promoter was enriched 3-fold compared with the input DNA, indicating direct binding of AmpR. This binding, although not as strong as AmpR binding at P*_ampC_*, is significant. Transcriptome and phenotypic data show that even at this reduced binding, AmpR is able to bring about significant changes in the QS system [Figure 8, (21)].

### Comparison of microarray and RNA-Seq

To get a comprehensive picture of the AmpR regulon in *P. aeruginosa*, we have performed transcriptomics studies using DNA microarrays (21) and RNA-Seq (this study). Both the transcriptomics studies were performed using the same two strains (PAO1 and PAOΔ*ampR*), under identical conditions (without and with sub-MIC β-lactam exposure).

The normalized data from the microarrays and RNA-Seq were compared to compute the extent of correlation. The Pearson correlation coefficients ranged from 0.31 to 0.70 for the four different conditions. Further, comparing the differentially expressed genes under the same four conditions showed correlation coefficients ranging from 0.66 to 0.99. This degree of correlation agrees with work done previously by ‘t Hoen *et al.* (93).

**Figure 11.**
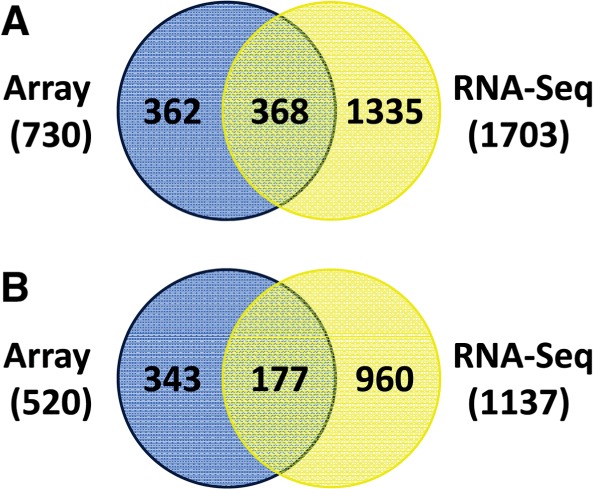
Comparative analyses of AmpR microarray and RNA-Seq datasets. All the differentially expressed genes (**A**) and the AmpR-regulated genes (**B**) from our previous AmpR microarray analysis (21) and the RNA-Seq data from the current study were compared.

Overlaps between the microarray and RNA-Seq datasets were determined for all the significantly differentially regulated genes in both PAO1 and PAOΔ*ampR* (Figure 11A). This revealed that deep sequencing identified 50% of the genes detected in the microarray (Figure 11A), with a correlation coefficient of 0.56 between the overlapping datasets. Examples of the overlapping genes have been discussed in the previous sections and include both positively regulated (numerous QS-regulated genes, *ampC*) and negatively regulated (*mexEF-oprN*, alginate regulators) genes. Moreover, no discordance was observed in the direction of fold change between the two techniques, and is in agreement with previous studies (94–96).

Comparing the AmpR-regulated genes between the microarray (21), and RNA-Seq datasets (this study) also revealed overlaps (Figure 11B). The genes identified in both the transcriptome studies to be AmpR-regulated include *ampC* (the primary target of AmpR) and genes of the type VI secretion system (*tssJ1, tssM1* and the *tse2-tsi2* operon). AmpR positively regulates the QS system [(21), this study]. Accordingly, the transcriptome studies identified AmpR-regulated QS genes such as *pqsE*, *hcnABC* (the hydrogen cyanide biosynthetic operon), *phz* (phenazine biosynthetic genes) and *mexGHI-opmD* (MexGHI-OpmD efflux pump).

Genes of the *mexEF-oprN* efflux pump are also downregulated in both of the transcriptome analyses (Figure 11B), and concurs with data from MIC studies (21). AmpR regulates genes present in RGPs by modulating expression of other transcriptional regulators (21). Accordingly, both microarray and transcriptome studies identified 13 genes in RGP03 and RGP04 to be differentially expressed in an AmpR-dependent manner.

Another interesting gene identified in both the microarray and the RNA-Seq analyses is *PA4378.* The *PA4378* gene is part of a three-gene operon (*PA4377–PA4379*). Both the array and RNA-Seq data showed increased expression of *PA4378* and *PA4379* in the absence of *ampR*. PA4378 has a 61% similarity to *E. coli* InaA (35). The expression of *inaA* is induced under stress conditions such as pH change in *E. coli* (35) and ice-nucleation in *Erwinia ananas* (97). In *E. coli, inaA* expression is regulated by SoxRS, the superoxide stress response system (98). The opposing regulatory effect on *inaA* expression (negative) and oxidative stress (positive) suggests that AmpR might regulate these two via SoxRS. However, there was no differential regulation of *soxRS* in the transcriptome studies. This interesting co-regulation of antibiotic resistance with oxidative stress response and the role of AmpR in this process need further investigation.

## DISCUSSION

In the opportunistic human pathogen *P. aeruginosa*, gene expression is a tightly controlled process with many regulators acting in concert to control virulence traits (11,12). *In silico* analyses and empirical evidence have identified critical regulators such as Vfr, the *P. aeruginosa* homolog of the *E. coli* cAMP receptor protein, to be central to *P. aeruginosa* pathogenicity (99–102). This study establishes the role of *P. aeruginosa* AmpR as not only a regulator of virulence factors, but also of important physiological processes such as response to oxidative stress and heat shock. In this study, we also identified sRNAs to be targets of AmpR regulation.

In the CF lung, chronic exposure to H_2_O_2_ released by polymorphonuclear leukocytes leads to *P. aeruginosa* overproducing the extracellular polysaccharide alginate (103). It has been established that the ECF sigma factor AlgT/U is the master regulator of alginate production by turning on constitutive expression of the *algD* operon during chronic infection (104). AmpR positively regulates the H_2_O_2_-mediated oxidative stress response, whereas negatively regulates *algT*/*U* expression in response to unidentified signals (22). Although it appears contradictory, AmpR is required for acute but not chronic infection, and thus it is expected to negatively regulate *algT/U* expression*.* Even though loss of *ampR* leads to enhanced-*algT/U* transcription, it does not translate into alginate production. This is due to posttranslational control of AlgT/U by its cognate anti-sigma factor MucA preventing AlgT/U-mediated *algD* transcription (105). Moreover, gene regulation is a complex interlinked process in *P. aeruginosa*, and multiple tiers of regulation for critical pathways are common (11,99,106).

**Figure 12.**
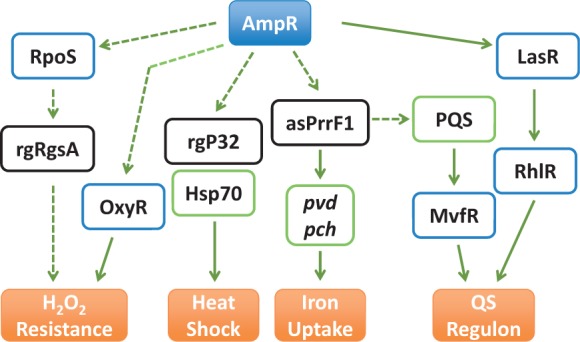
AmpR-mediated regulation of virulence and physiological processes in *P. aeruginosa*. AmpR affects expression of QS genes by directly binding to P*_lasR_* and modulating *lasR* expression. AmpR also positively regulates the PQS by modulating levels of the antisense RNA asPrrF1, thus also affecting iron uptake. By regulating expression of the stationary-phase sigma factor RpoS (21), *oxyR* and the small RNA rgRgsA, AmpR positively regulates the oxidative stress response. The genes encoding the Hsp70 heat-shock response system are also positively regulated by AmpR. This and previous findings (21) demonstrate that AmpR is a major regulator of virulence and physiological processes in *P. aeruginosa*.

AmpR is required for bacterial survival upon heat shock. However, AmpR did not regulate expression of *rpoH,* encoding the heat-shock sigma factor (data not shown). This is not surprising because expression of many sigma factors, including RpoH, is regulated post-transcriptionally (58,59). However, high-temperature survival may be mediated by small regulatory RNA rgP32, DnaJ and DapB (35), the members of Hsp70 heat-shock response system (54) that are positively regulated by AmpR (Figure 12).

Iron acquisition is a critical determinant of *P. aeruginosa* pathogenicity and has been proposed as a potential target to counter infections (107). In healthy lungs, free iron is available only in very low quantities, as it is typically bound by ferritin and transferrin (108). In the CF lungs, however, higher free iron concentration is attributed to successful colonization by bacteria such as *P. aeruginosa* (108). *Pseudomonas aeruginosa* uses siderophores pyoverdine and pyochelin to sequester extracellular iron under limiting conditions (109,110). In addition, the *P. aeruginosa* PQS helps in iron chelation, facilitating the activity of pyoverdine and pyochelin (111–113). AmpR positively regulates expression of both siderophores and PQS genes, and the loss of *ampR* results in growth impairment under iron-limited conditions (Figure 4). However, iron is not a limiting factor in the CF lung (108,114). Thus, the role of AmpR in iron uptake in the CF lungs needs further investigation.

AmpR positively regulates the stress-specific sigma factor RpoS and RpoS-regulated virulence factors in a growth-phase dependent manner (21). So, a subset of the AmpR-regulated genes is possibly regulated via RpoS. As the link between QS and RpoS is well established (115,116), AmpR regulation of both these processes is not surprising (Figure 12). RpoS positively regulates expression of the sRNA *rgsA* (*PA2958.1*) that contributes to H_2_O_2_ resistance in *P. aeruginosa* (63). GacA positively regulates rgRgsA (63). In this study, AmpR positively regulates RpoS, GacA and rgRgsA (Figure 12), as well as OxyR, the major regulator of the catalase KatA that ultimately affects resistance to H_2_O_2_ (Figure 12). GacA affects QS signaling by controlling the expression of RsmA, a negative regulator of the *las* QS system (83,88,117,118).

Iron uptake, QS and oxidative stress are positively regulated by AmpR. However, these phenotypes appear disparate but they are, in fact, interlinked [Figure 12, (11)]. The relationship between iron uptake and QS is complex, and some of the transcriptional regulators (such as MvfR) involved in QS regulation also modulate iron response (119–121). The iron-responsive sigma factor PvdS (PA2426) turns on *mvfR* transcription in response to iron starvation (122,123). In addition, the antisense RNAs, asPrrF1 and asPrrF2, positively regulate production of the PQS signaling molecule (124). Our data demonstrate that AmpR positively regulates expression of *pvd* (pyoverdin genes), *pch* (pyochelin genes), *mvfR* and *prrF1* but not *prrF2*, thus affecting iron uptake and PQS synthesis. Of the 15 genes in the rgPrrF1 regulon (125), AmpR regulates positively *PA2514* and *PA4236,* whereas negatively *PA1581 and PA3531*. Both *PA2514* and *PA1581* are part of two distinct operons (35). So AmpR could also potentially regulate other genes in the operon, but were not detected in our assay. However, it is tempting to speculate that these genes are regulated in an AmpR-independent manner. This manner of multiple tiers of gene regulation is not surprising in *P. aeruginosa* (11,12).

CF lung isolates are genotypically and phenotypically heterogeneous [reviewed in (126)]. Higher mutation rates are the driving force behind the *P. aeruginosa* population heterogeneity (127–129). Specifically, mutations in *mucA* and *lasR* arise early in the colonization process, followed by mutations in anti-mutator genes such as *mutS*, *mutT*, *mutY* and *mutM* (130). MucA mutations trigger the regulated intramembrane proteolytic cascade, freeing AlgT/U and allowing for overexpression of alginate [reviewed in (131)]. Mutations in *lasR* would abolish expression of QS-regulated acute virulence factors [reviewed in (132)]. In the CF lung, *P. aeruginosa* loses the ability to produce acute virulence phenotypes after initial colonization and starts to overexpress chronic infection traits (133). AmpR mutant strains display some characteristics reminiscent of CF isolates, including acquisition of fluoroquinolone resistance, reduced production of QS-regulated virulence factors such as proteases and pyocyanin and enhanced biofilm formation [this study, (21)]. Furthermore, loss of *ampR* leads to increased expression of *mutY* (*PA5147*; 2.4-fold, *P* 9.25E-06) and *mutM* (*PA0357*; 2.6-fold, *P* 5.28E-06) when exposed to β-lactam (Supplementary Table S4), indicating negative AmpR regulation. So, one would expect the mutation frequencies to decrease in the absence of *ampR*, given that some anti-mutators are overexpressed. However, loss of *ampR* did not result in significant change in mutation frequencies for rifampicin and streptomycin (data not shown). This seeming contradiction is not surprising because MutY and MutM are known to be weak anti-mutators in *P. aeruginosa*, unlike MutS (130). These data suggest that inactivating *ampR* in the CF lung, in addition to other mutations, will help *P. aeruginosa* colonize better. However, the occurrence and frequency of *ampR* mutations in CF isolates needs to be determined.

Our previous and current analyses showed that AmpR positively modulates LasR, affecting QS phenotypes [Figure 8, (21)]. ChIP-Seq and complementary data demonstrated that AmpR directly binds to P*_lasR_*. AmpR-binding site analysis using the ChIP-Seq data revealed a motif that was very similar to that identified in our previous studies using microarrays (21). Minor motif variations from the consensus sequence are expected. A consensus sequence arrived using data from multiple promoters is likely to be more accurate as compared with footprinting studies that look at individual promoters. The AmpR motif identified in this study (Figure 10) using ChIP-Seq data appears to be more refined as compared with previous analysis (21). This motif is likely to closely resemble the AmpR-binding site, as it was determined based on multiple promoters.

In conclusion, the data presented here, and previously by our laboratory, demonstrate that *P. aeruginosa* AmpR is a critical component in regulating virulence, metabolism and physiological processes. The clinical significance is highlighted in a recent study on extremely drug-resistant high-risk *P. aeruginosa* hospital isolates that harbor constitutive AmpR-activating mutations leading to AmpC overproduction (134). Moreover, given the global regulatory effect, it is likely that other virulence traits exhibited by high-risk clinical isolates (134) are also AmpR-mediated. Small molecule inhibitors targeting specific proteins such as *P. aeruginosa* NagZ (135) and *Vibrio cholera* LuxO (136) show therapeutic promise. Inhibitors of AmpR function will render the strain sensitive to β-lactam antibiotics and reduce the production of acute virulence factors. Thus, combination therapies using AmpR inhibitors and antibiotics will potentially provide us with means to counter *P. aeruginosa* infections, and warrant further investigation.

## SUPPLEMENTARY DATA

Supplementary Data are available at NAR Online.

## FUNDING

National Institutes of Health-Minority Biomedical Research Support SCORE grants [S06 GM08205, 5SC1AI081376 to K.M.]; Florida Department of Health [09KW-10 to G.N. and K.M.]; Florida International University (FIU) Research Assistantship (Herbert Werthiem College of Medicine, to D.B.); FIU College of Computing and Information Science Post Doctoral Fellowship (to D.B.); and FIU University Graduate School Dissertation Year Fellowship (to D.B.). Funding for open access charge: College of Engineering and Computing, Florida International University (to G.N.).

*Conflict of interest statement*. None declared.

## Supplementary Material

Supplementary Data
