# Supplementary material for: Deep sequencing analyses expands the Pseudomonas aeruginosa AmpR regulon to include small RNA-mediated regulation of iron acquisition, heat shock and oxidative stress response
Source: Nucleic Acids Res. 2013 Oct 23;42(2):979–98. doi: 10.1093/nar/gkt942 (PMC3902932; doi:10.1093/nar/gkt942)
Supplement: Supplementary Data [file supp_42_2_979__index.html]

Deep sequencing analyses expands the Pseudomonas aeruginosa AmpR regulon to include small RNA-mediated regulation of iron acquisition, heat shock and oxidative stress response — Deep sequencing analyses expands the Pseudomonas aeruginosa AmpR regulon to include small RNA-mediated regulation of iron acquisition, heat shock and oxidative stress response — Supplementary Data 

# Deep sequencing analyses expands the *Pseudomonas aeruginosa* AmpR regulon to include small RNA-mediated regulation of iron acquisition, heat shock and oxidative stress response

## Supplementary Data

files

**Files in this Data Supplement:**

- Supplementary Data - xlsx file
